# Systematic review of interventions to reduce ethnic health inequalities in maternal and perinatal health in the UK

**DOI:** 10.1136/bmjph-2024-001476

**Published:** 2025-07-15

**Authors:** Oluwaseun B Esan, Nicholas K Adjei, Samira Saberian, Lara Christianson, Alya Mazlan, Rukun K S Khalaf, Olukemi Yetunde Towolawi, Philip McHale, Andy Pennington, Rebecca Sally Geary, Abimbola Ayorinde

**Affiliations:** 1 Department of Public Health Policy and Systems, University of Liverpool, Liverpool, UK; 2 Library, Leibniz Institute for Prevention Research and Epidemiology, Bremen, Germany; 3 Academic Research, Independent Researcher, Oshodi, Lagos State, Nigeria; 4 Division of Health Sciences, Warwick Medical School, University of Warwick, Coventry, UK

**Keywords:** sociodemographic factors, systematic review, public health, social medicine

## Abstract

**ABSTRACT:**

**Introduction:**

There are persistent ethnic health inequalities in maternal, neonatal and infant health outcomes in the UK. We sought to examine the available evidence on interventions to reduce ethnic health inequalities in maternal, neonatal and infant outcomes during pregnancy and up to the first year of the postnatal period.

**Method:**

We conducted a systematic review searching MEDLINE, CINAHL, PsycINFO, Scopus and Web of Science (Social Science Index) databases, *Journal of Health Visiting*, Google Scholar and grey literature from relevant websites (from inception up to 11 August 2023). Interventions were mapped to a priori conceptual framework consisting of six levels (patient, provider, microsystem, organisation, community and policy). The ‘template for intervention description and replication’ checklist was used for intervention description. Results across studies were narratively synthesised and reported following the ‘synthesis without meta-analysis’ guidelines.

**Results:**

The electronic search identified 11 600 studies, with 16 studies describing eight types of interventions meeting the inclusion criteria. Studies were published between 1981 and 2022, predominantly in England (n=14), with a range of outcomes reported, including mode of delivery, place of birth, birth weight, stillbirth and preterm birth. The sample size varied from 21 to 20 651 participants with ethnic minority populations ranging from 18.9% to 100% of the study population. Studies mapped mainly to the patient level with policy least represented (14 and two, respectively). All studies described the reasons for the intervention with limited reporting on any modification during the study (n=2). Two studies with two types of interventions (early pre-eclampsia screening and midwifery continuity of care) demonstrated the potential for interventions to reduce ethnic health inequalities.

**Conclusion:**

This review highlights the paucity of evaluated interventions to tackle ethnic health inequalities in maternal, neonatal/infant outcomes. Mapping interventions to the conceptual framework provides the evidence base for national policy interventions to tackle these long-protracted inequities.

**PROSPERO registration number:**

CRD42023453083.

**WHAT IS ALREADY KNOWN ON THIS TOPIC**

- Ethnic inequalities in maternal and neonatal outcomes occur throughout pregnancy and the postpregnancy period up to 1 year following pregnancy.
- Systematic reviews providing evidence on interventions to reduce ethnic health inequalities do not cover the entire spectrum of pregnancy to early motherhood and neonatal/infant health.

**WHAT THIS STUDY ADDS**

- Only midwifery continuity of care operated at the policy level, offering comprehensive system-wide potential to reduce ethnic health inequalities; however, setbacks in roll-out, such as inadequate staffing levels, have halted progress.
- There is an overwhelming focus on interventions targeting patients, which promotes the broken bodies discourse, with a lack of focus on key outcomes for measuring progress in reducing ethnic health inequalities.

**HOW THIS STUDY MIGHT AFFECT RESEARCH, PRACTICE OR POLICY**

- Future intervention studies on ethnicity and maternal and neonatal health in the UK should use robust theoretical frameworks, such as the modified Clarke conceptual framework in this study, to highlight the main levers of change.
- Studies should provide clear disaggregated ethnicity classification, with a baseline measurement of ethnic health inequalities, and specifically state the objectives of reducing ethnic health inequalities.
- A portal of evaluated and ongoing interventions is warranted to reduce waste across the healthcare system and amplify examples of good practice.

## Introduction

 Ethnic inequalities in maternal, neonatal and infant healthcare are systematic differences in health outcomes and healthcare experiences among different ethnic or racial groups. These inequalities are unfair and avoidable as they are not linked to constitutional factors such as genetics but are predicated on the structuring of society.^1^ In the UK, these inequalities have been well documented with limited improvement since the publication of the first Confidential Enquiry into Maternal and Child Health in 2007.^2^

The most recent Mothers and Babies: Reducing Risk through Audits and Confidential Enquiries across the UK report found that Black women have nearly four times the maternal mortality rate of White women during pregnancy or up to 6 weeks after childbirth or the end of pregnancy.^3^ There is also variation within broad ethnic categories that points to societal causes; for instance, within the broad Asian category, Indian mothers and infants had a lower rate of adverse outcomes in comparison with Bangladeshi or Pakistani mothers and infants.^3 4^ Migration also plays a role, as asylum seekers and refugees are known to experience adverse outcomes due to inadequate access to healthcare.^5^

Policy efforts to address ethnic inequalities in maternal, neonatal and infant health outcomes in the UK span four decades.^6^ Despite this, translating policy into improved frontline care and outcomes for minority groups remains challenging.^6^,^8^ Previous evidence syntheses have largely focused on service access and experiences, with limited evidence on interventions that improve health outcomes.^9^ Existing evidence of improved outcomes concentrates primarily on antenatal interventions, lacking coverage of the full maternal and neonatal care spectrum in the UK.^10 11^ The 2021 census recorded 287 ethnic groups, with minority populations increasing since 2011—showing 81.7% White, 10.1% mixed, 9.3% Asian, 2.5% Black and 1.6% other groups, highlighting the imperative to address ethnic health inequalities.^12^

COVID-19 highlighted stark health inequities in the UK, with Black, Asian and mixed-ethnic groups facing disproportionately high morbidity and mortality.^1 13^ These inequities reflect long-standing issues, including poorer maternal and infant outcomes such as higher maternal mortality and lower birth weight.^1 14^ In response, the NHS Race and Health Observatory commissioned a scoping review to map policy interventions addressing ethnic health inequalities in England.^15^ While the previous review focused solely on England, there is a need to examine evidence across all UK nations—England, Scotland, Wales and Northern Ireland, despite devolved healthcare administration.

This systematic review extends the previous scoping review with a focus on outcomes reported by the National Maternity and Perinatal Audit (NMPA). The NMPA is a large-scale audit of National Health Service (NHS) maternity services across England, Scotland and Wales.^16^ It aims to evaluate a range of care processes and outcomes to identify good practices and areas for improvement in the care of women and babies. Although Northern Ireland (a constituent of UK countries) is not included in the NMPA, it is crucial to assess interventions with clear reporting of outcomes in measuring the reduction in ethnic health inequalities in outcomes as covered by the NMPA.^16^ Hence, the aim of this review is to examine the available evidence on interventions to reduce ethnic health inequalities in maternal, neonatal and infant outcomes during pregnancy and up to the first year of the postnatal period in the UK.

## Method

### Search strategy and selection criteria

The protocol for this systematic review was developed prospectively and registered before any stage of the systematic review was completed (PROSPERO Unique Identification number CRD42023453083).^17^ The Preferred Reporting Items for Systematic Reviews and Meta-Analyses guidelines were followed.^18^

We updated and expanded our previous searches for a scoping review on interventions to tackle ethnic health inequalities in maternal and perinatal outcomes in England^15^ to cover all constituent countries of the UK. The following electronic bibliometric databases were searched from inception until 11 August 2023: MEDLINE (OvidSP) (1946–present), PsycINFO (OvidSP) (1806–present), Scopus (1981–present), Web of Science Social Science Citation Index (1900–present) and CINAHL (EBSCOHost) (1981–present). We also searched the online version of the *Journal of Health Visiting* as this journal is not indexed in the selected databases. The electronic searches were supplemented by hand searching, examining reference lists of key papers and reports and identifying grey literature from websites of organisations such as the National Perinatal Epidemiology Unit, relevant third sector organisations such as the Pregnancy and Baby Charities Working Together, Maternity Action, Tommys, Sands, Best Beginnings, as well as searches conducted via Google Scholar.

The screening was conducted in a two-stage process. Due to the high volume of records, all titles and abstracts were screened based on inclusion and exclusion criteria. Seven reviewers were involved in the first stage of screening (OBE, AM, RKSK, SS, AA, PMcH, OYT) and 20% of the excluded articles were checked by a second reviewer (NA, PMcH, AM). For the second stage, all full-text screenings were independently double-screened by two reviewers (OBE and AM, RKSK and OYT, SS and OYT) with discrepancies resolved by a consensus with the lead reviewer (OBE).

The search strategy was developed by a team of systematic review experts and information scientists (LC, AP). It included thesaurus, free-text terms and relevant synonyms for the population (pregnant women, babies, ethnicity/disaggregated ethnic groups), intervention and outcome levels of the inclusion criteria and used proximity operators where appropriate (see online supplemental file 1). The search terms were combined using appropriate Boolean operators. To ensure all study types were captured, we did not use methodological filters, but we applied the MEDLINE filter for the UK.^19^ We did not restrict our searches by year of publication as a pilot search did not indicate a significant reduction in results. The final searches were imported into Rayyan.^20^

The screening was conducted as a two-stage process. First, all titles and abstracts were screened based on the inclusion and exclusion criteria, followed by a full-text screening. The first stage was carried out independently by seven reviewers (OBE, AM, RKSK, SS, AA, PMcH, OYT); three reviewers (NA, PMcH, AM) reviewed 20% of all excluded articles. For the second stage, full-text screening was also independently double-screened by five reviewers (OBE, AM, RKSK, SS, OYT), and discrepancies were resolved by consensus with the lead reviewer (OBE).

### Inclusion and exclusion criteria

The systematic review included studies that evaluated interventions for maternal and perinatal health by ethnic categories based on the outcomes reported in the NMPA as defined by the Population, Intervention, Comparator and Outcomes framework. The population of interest included any mothers, pregnant women and babies up to age 1 with any intervention in any healthcare setting, with or without a comparator with outcomes reported for at least one ethnic minority group (online supplemental file 2). We focused on literature in the UK without restriction on the period to capture previously effective interventions that may no longer be widely used (online supplemental file 2). We excluded observational studies that focused on health outcomes (eg, morbidity, mortality) without intervention and also excluded studies that reported only ‘experiences or access’ of/to services for ethnic minority groups without interventions (online supplemental file 2).

### Risk of bias

We appraised the quality of included studies using the Mixed Methods Appraisal Tool (MMAT).^21^ This tool is a standardised instrument developed by McGill University, Canada, for systematic reviews. The MMAT includes five quality appraisal criteria for each of the five study designs, including qualitative studies, randomised controlled trials, non-randomised studies, quantitative descriptive studies and mixed methods studies. Quality assessment was performed by two reviewers (OBE and NA) following a calibration exercise with two studies. The completed quality assessments were cross-checked, and discrepancies were resolved through a consensus.

### Data extraction and analysis

The review team developed a prepiloted data extraction form in Microsoft Excel, including four main sections: general information, checklist items, intervention information and outcomes by ethnicity and mapping to the conceptual framework (online supplemental file 3). Reviewers OBE and NA, OBE and AM, SS and NA, double-extracted data from studies into the form. An online interactive table—Airtable was used for intervention description according to the Template for Intervention Description and Replication (TIDieR).^22^ Reviewers SS and PMcH, NA and AA, AA and NA, OYT and SE, AM and SE worked in pairs, with one reviewer extracting the data and the other reviewer checking the extracted data. The lead reviewer, OBE, checked all extracted data to ensure consistency of reporting. The guideline for a ‘systematic review without meta-analysis (SWIM)’^23^ was adopted for the narrative synthesis due to anticipated heterogeneity across studies. The tables and literature were synthesised according to the type of intervention(s) and mapped to a conceptual framework from a previous scoping review (online supplemental file 4).^15^ Ethnicity was reported according to the authors’ categorisation and groupings in the published tables. The potential for interventions to reduce health inequalities was assessed using harvest plots based on vote counting, with evidence certainty evaluated through risk of bias and directness to the research question.^23^

### Role of the funding source

The funder of the study had no role in study design, data collection, data analysis, data interpretation or writing of the report. All researchers were independent of the funders and all authors had access to the data and accepted responsibility for the decision to submit for publication.

### Patients and public involvement

No patient and public involvement.

## Results

### Study selection and inclusion

**Figure 1:**
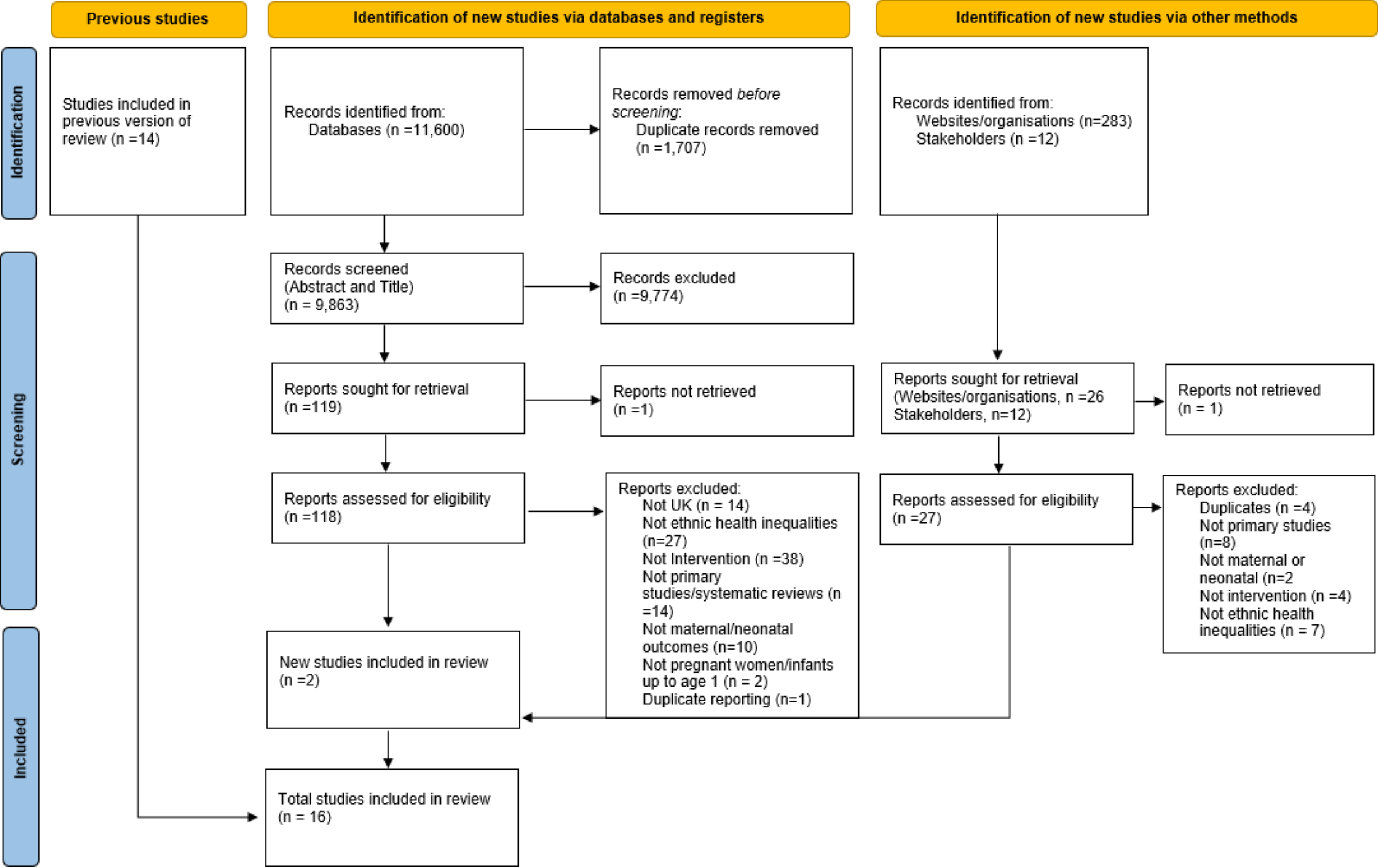
Preferred Reporting Items for Systematic Reviews and Meta-Analyses flow diagram of included studies.

The electronic database search identified 11 600 titles for initial screening. After removing duplicates (n=1707), 9893 studies remained for abstract examination. 9774 were excluded after abstract examination. A total of 119 published full-text studies were assessed for eligibility (figure 1). Studies were excluded if they were not an intervention (n=38), did not report outcomes by ethnicity/not ethnic health inequalities (n=27), were not a primary study or was a review (n=14), conducted outside of the UK (n=14), no reported maternal or perinatal outcomes (n=10), population was not pregnant women/mothers/infants up to age 1 (n=2), duplicate publication (n=1) or could not be retrieved (n=1) (figure 1 and online supplemental file 5). Finally, a total of 16 peer-reviewed studies (14 from previous review) were included in the systematic review.

### Characteristics of included studies

The characteristics of the included 16 studies are listed in table 1.

**Table 1:** Characteristics of included studies

Study	UK/NHS England Region	Study design; data source	Sample size (age in years); (dates data collection)	Ethnic minority group (% of total sample)	Population	MMAT score
Antenatal and postnatal education						
McEnery 1986^25^	London, England	Cohort	69 (18–40); (1980–1982)	Pakistani, East African Asians	Mothers and infants	*
Brookes 2015^37^	West Midlands, England	Qualitative; primary data collection	14 parents (three fathers, 11 mothers)	Pakistani, Indian, Chinese, Oromo (cultural group in Ethiopia), Somali, Bangladeshi, Caribbean (St Vincent)	Parents	****
Antenatal screening						
Dormandy 2010^28^	London, England	RCT; other	1454 (>18); (2005–2006)	North European, South or Southeast	Pregnant women and fathers	***
				Asia, African/Caribbean, South European, other and mixed; (60.3%)		
Liu 2022^31^	London, England	Cohort; hospital records	20 651 (NICE: 32.6 (29.2–35.9); FMF 32.8 (29.4–35.9)); (2016–2020)	White, Black, Asian and mixed/other; (34.4%)	Pregnant women	***
Health advocacy/lay support/link worker						
Mason 1990^35^	East Midlands, England	Case-control; primary data collection	485 (NR); (1985–1986)	Asian women; (100%)	Pregnant women, mothers and babies	****
Parsons 1992^26^	London, England	Unknown; hospital records	1000 (NR); (1986)	Born in Asia or Turkey; (100%)	Pregnant women	*
Smith 2004^36^	East of England, England	Qualitative; other	30 (3 months); (2000)	British Pakistani; (100%)	Babies	*****
Wiggins 2005^27^	London, England	RCT; primary data collection	731 (NR); (1999)	Women living in deprived areas Camden and Islington; (42.5%)	Mothers	****
Yuan 2010^34^	Belfast, Northern Ireland	Quasi-experimental	32 (NR); (NR)	Chinese (100%)	Mothers	***
Interpreter services						
Barnes 2011^38^	England	Mixed methods	1304 (<16–24); (2007–2009)	White, Black, Asian, mixed or other (18.9%)	Mothers, nurses and interpreters	**
Midwifery continuity of care						
Homer 2017^29^	London, England	Cohort; primary data collection	2568 (14–50); (1997–2009)	Black African, Black Caribbean, Black British, Asian, mixed, other and unknown (63.1%)	Mothers and babies	****
Sioti 2020^39^	Yorkshire & The Humber	Mixed method evaluation	21 (18–34); (February 2018–February 2019)	Democratic Republic of Congo, Eritrea, India, Iran, Kurdistan, Kuwait, Nigeria, Pakistan, Saudi Arabia, Sri Lanka, Sudan, Syria, Yemen	Mothers and infants	****
Hadebe 2021^30^	London, England	Cohort; hospital records	LEAP area caseload: 230 pregnancies, LEAP traditional care: 293, non-LEAP all care: 8430 pregnancies (NR); (2018–2020)	White, Black, Asian, mixed, other; (LEAP—47.8%, LEAP traditional—42.8%, non-LEAP all care—37.8%)	Mothers and babies	*****
Perinatal mental health intervention						
Husain 2023^32^	North West	RCT; primary data collection	83 (>16); ()	Indian, Pakistani, Bangladeshi; (100%)	Mothers	***
Vitamin D						
Maxwell 1981^24^	London	RCT; primary data collection	126 (NR); (NR)	Asian; (100%)	Pregnant women	****
Datta 2002^33^	Wales	RCT; primary data collection	160 (NR); (1995–1996)	100 from Indian subcontinent, four were Afro-Caribbean, nine from Middle East, 11 from Far East, 36 from Africa	Pregnant women	****

Star rating used to prevent from a focus on a single score.^21^ Information on each intervention is available in online supplemental file 6.

FMF, Fetal Medicine Foundation; LEAP, Lambeth Early Action Partnership; MMAT, Mixed Methods Appraisal Tool; NHS, National Health Service; NICE, National Institute for Health and Care Excellence; NR, not reported; RCT, randomised controlled trial.

Studies were published between 1981 and 2023 with the majority conducted in England (87.5%, 14/16), of which half were conducted in London (50%, 7/14).^24^,^31^ The study size varied from 21 to 20 651, with the proportion of ethnic minorities ranging from 18.9% to 100%. Participants included pregnant women, mothers, babies, family members, midwives and other health professionals. Twelve studies adopted a quantitative design (randomised controlled trial (RCT),^24 27 28 32^ cohort,^2526 29^,^31 33^ quasi-experimental^34^ and case-control^35^), two studies were qualitative (interviews,^36^ focus group^37^) and two were mixed methods.^38 39^ Of the 12 quantitative studies, the majority reported a comparator (91.6% 11/12),^24^,^2830^ various maternal and neonatal outcomes were reported (place of birth,^29 30^ mode of delivery,^26 29 30 39^ stillbirth,^29^,^3139^ birth weight,^24 26 35^ preterm birth,^29^,^3139^ maternal vitamin D levels^24 33^ and infant vitamin D levels^33^) and over a third adjusted for confounders such as English proficiency,^30 32 35^ comorbidity,^30^ unknown ethnicity,^30^ ethnicity,^27^ mother’s education^27^ and level of deprivation^27^ (41.7%, 5/12). Of the remaining eight studies without adjustment for confounders,^24^,^2628 29 31 33 34^ two reported baseline characteristics of potential confounders^29 34^ (table 1 and online supplemental file 6).

### Completeness of intervention reporting and quality appraisal

All 16 studies addressed the questions in the TIDieR checklist (online supplemental file 7) to varying levels of completeness. All studies reported why they were carrying out the intervention, what the intervention was and who provided the intervention (n=16). The least reported areas were on tailoring of interventions (n=9), information about intervention fidelity (n=6) and modification (n=2).

The quality of the studies varied across the different study designs (online supplemental file 8). The four RCTs all achieved 60% of the MMAT quality criteria.^24 27 28 32^ Missed quality criteria included a lack of comparable groups at the baseline,^28^ complete outcome data, blinding of outcome assessors to the intervention^27 28^ and lack of clarity on participants’ adherence to the assigned intervention.^24 32^ The seven cohort and case-control studies^2526 29^,^31 33 35^ achieved MMAT quality ranging from 20% to 100%. The least reported quality criterion was adjustment for confounding (only three studies),^30 33 35^ which may limit the inference of the estimates. Of the two qualitative studies,^36 37^ 80% and 60% of the quality criteria were reported. For the remaining two mixed methods studies,^38 39^ one achieved 80% of the quality criteria^39^ and the other just 60%^38^ (online supplemental file 8).

### Characteristics of the interventions

**Figure 2:**
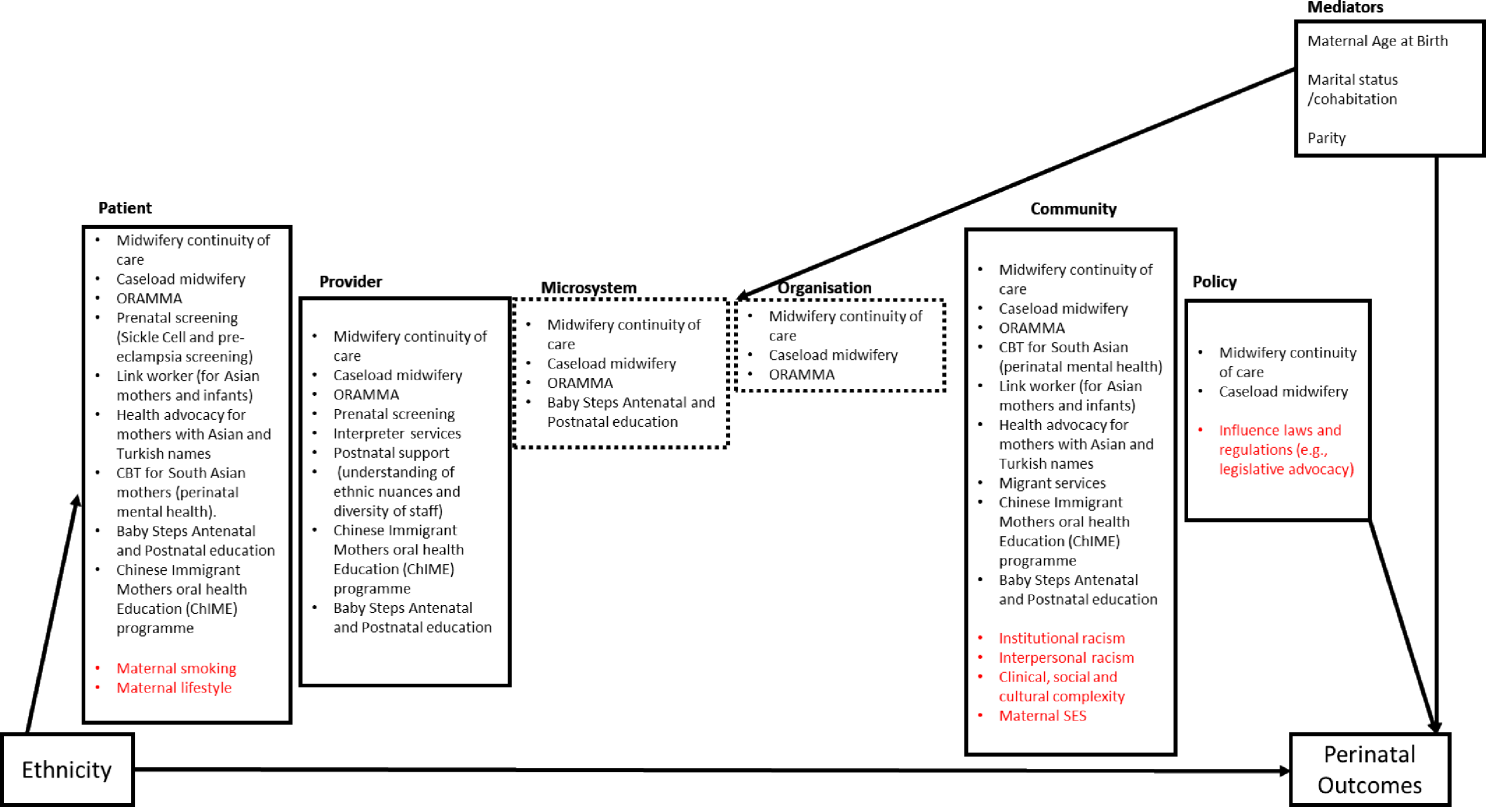
Identified interventions mapped to the conceptual model. The figure shows an a priori conceptual framework adapted from the Clarke review to map identified interventions in Black populations. It considers a causal framework by considering how structural factors shape ethnicity, which can directly affect patients and influence outcomes across all levels. The framework also includes mediators—factors that can impact perinatal outcomes. The red items represent missed opportunities. This framework was informed by evidence on the levels of patient care that are amenable to interventions aimed at reducing ethnic health inequalities in perinatal outcomes.^45^^65^ CBT, cognitive behavioural therapy; ORAMMA, Operational Refugee and Migrant Mothers Approach (midwifery continuity of care for refugee and asylum seekers); SES, socioeconomic status; Baby Steps is an antenatal and postnatal educational programme.

The 16 studies covered all levels of the a priori conceptual framework and could be mapped to multiple levels within it (figure 2). Fourteen of the studies identified mapped to the patient level of the framework^24^,^2628^ and aimed to improve maternal physical health,^24 29 30 33 35 39^ maternal mental health,^32^ neonatal health^2428^,^31 33 39^ and infant health.^25 34 36 37^ Seven of the studies mapped to the provider level to improve the quality of care provided, including reducing language barriers.^27^,^3037^ Four studies mapped to the microsystem affecting the immediate care team, such as a change in the shift pattern^29 30 37 39^; three studies mapped to the organisation level requiring a change in service delivery^29 30 39^; eight studies mapped to the community level to improve access to healthcare and address social/cultural complexity in the delivery of interventions.^2629 30 32 35^,^37 39^ Only two studies mapped to the policy level requiring resource allocation at a national level.^29 30^ The studies were further categorised into seven main groups (antenatal and postnatal education; health advocacy/lay support/link worker; interpreter services; midwifery continuity of care (MCoC) models; perinatal mental health; prenatal/neonatal screening and vitamin D supplementation) according to the main elements of the interventions (online supplemental file 9).

#### Antenatal and postnatal education

Two studies aimed to improve maternal and infant health outcomes; one among Pakistani and East African Asian mothers in London^25^ and ‘Baby Steps’, an educational programme for multiple ethnic groups (Pakistani, Indian, Chinese, Oromo, Somali, Bangladeshi and Caribbean).^37^ The London study measured maternal nutrient levels,^25^ while Baby Steps assessed knowledge, confidence and family relationship outcomes.^37^ The interventions targeted the patient,^25 37^ provider,^37^ microsystem^37^ and community^37^ levels of the framework. Baby Steps showed promise in reducing health inequities through improved pregnancy and parenting knowledge, enhanced relationships and positive attitudinal changes regarding gender roles, corporal punishment and female genital mutilation (online supplemental file 9).

#### Antenatal screening

Two studies reported outcomes on antenatal screening.^28 31^ One evaluated outcomes of early screening for sickle cell using a cluster RCT (n=1454),^28^ and another assessed the impact of first-trimester screening for pre-eclampsia and stillbirth, neonatal and perinatal death rate in a large retrospective cohort study (n=20 561).^31^ These interventions targeted the patient level of the framework, with the early screening for sickle cell also targeting the provider level.^28^ Uptake for early screening for sickle cell was low, hence the results could not be disaggregated by ethnic categories,^28^ while the Fetal Medicine Foundation (FMF) first-trimester screening for pre-eclampsia in comparison with the standard screening according to the National Institute for Health and Care Excellence (NICE) reported a reduction in preterm birth rate for non-White women in comparison with White women. White women (NICE screened vs FMF screened, OR 0.969 (95% CI 0.493 to 1.908)), non-White women (NICE screened vs FMF screened, OR 0.403 (95% CI 0.206 to 0.789)). Although these studies achieved a high-quality rating of over 80%, only the first-trimester screening study specifically stated the aim of reducing ethnic inequalities in its objectives^31^ (online supplemental file 8).

#### Health advocacy/lay support/link worker

Five studies examined non-health professionals in supporting mothers at different stages of motherhood from the antenatal^26 35^ to the postnatal period^27 34 36^ using RCT,^27^ observational,^26 35^ quasi-experimental^34^ and qualitative study^36^ designs (table 1 and onlinesupplemental files 6^ 9^). Study sizes were small, with two studies having fewer than 40 participants^34 36^ and the remaining studies between 400 and 1000.^26 27 35^ Four studies focused on the same ethnic group—Asian,^26 35^ South Asian^36^ and Chinese,^34^ while a fifth study did not list the ethnic minority groups of participants but indicated the proportion who needed interpreters and who were from a minority ethnic group.^27^ Support workers were usually from the same ethnic/cultural background as participants,^2634^,^36^ and sometimes provided an interpreter service.^26^ Outcomes measured included birth weight,^35^ care processes (proportion of antenatal length of stay,^26^ caesarean, induction^26^ and mode of delivery^26^), behavioural indicators (maternal smoking,^27^ weaning at age 1^36^) and psychological factors (maternal depression,^27^ mother-infant bonding index).^34^ These interventions targeted the patient^2634^,^36^ and community levels of the framework.^27^ Study quality varied considerably (MMAT 10%–80%), with only three studies explicitly stating the aim to improve outcomes for specific ethnic groups.^34^,^36^ The remaining studies either evaluated improvement in maternal and perinatal outcomes in all women,^27^ while another study used the evaluation of health advocacy for Asian women as a proxy to measure the influence on hospital policy and practice without specifically outlining the aim of reducing ethnic health inequalities in the objectives.^26^

#### Interpreters

One study evaluated the role of interpreters in delivering health visits to teenage mothers.^38^ The reported outcomes included key delivery indicators of the nursing partnership by health visitors, such as the percentage of planned content covered, the percentage of time spent on five content domains and the client’s understanding and involvement^38^ (online supplemental file 9).

#### Midwifery continuity of care models

Three studies evaluated the role of having the same midwife or team of midwives from antenatal to postnatal period (table 1).^29 30 39^ Two of the studies were conducted in the same part of South London, England, with over a third of the participants experiencing high levels of social deprivation.^29 30^ Homer et *al* showed that Black, Asian and minority ethnic women were more likely to have preterm or low birthweight babies than White women (Black, Asian and minority ethnic women vs White women: preterm birth (<37 weeks) 100 (6.2%) vs 30 (3.2%) p≤0.001; low birth weight (<2500 g) 90 (6.1%) vs 28 (3.2%) 0.002), but with a lack of a comparator group between those who experienced MCoC versus standard care. While Hadebe *et al* demonstrated a significant reduction in the rate of caesarean section, Black, Asian and minority ethnic women (43.1%–27.8%); risk ratio 0.68 (0.47 to 0.99) p=0.04 vs White women (39.8%–24.7%); risk ratio 0.63 (0.40 to 0.99) p=0.04 compared with standard care. The third study was conducted in Sheffield, England, as part of a European-wide study on MCoC for asylum-seeking and refugee women.^39^ The midwives, maternity peer supporters as well as migrant, asylum-seeking and refugee women all found the culturally appropriate and individualised care provided by the multidisciplinary team improved early antenatal booking and engagement. These interventions targeted all levels of the conceptual framework and achieved a good MMAT quality rating (60%–80%). One study demonstrated the potential to reduce ethnic health inequalities, with inference in the remaining two studies limited by the lack of a comparison group^29^ (online supplemental file 9).

#### Perinatal mental health

A single RCT (n=83) evaluated culturally adapted CBT for postnatal depression among South Asian mothers (Indian, Pakistani and Bangladeshi) in Manchester and Lancashire.^32^ Despite good methodological quality (MMAT 60%), the patient and community-level intervention showed no significant improvement in depression scores compared with usual treatment, possibly due to limited sample size and geographical coverage.^32^

#### Vitamin D supplementation

Two studies assessed the impact of vitamin D supplementation in one RCT of Asian women in London, England published in 1981^24^ and one cohort study of women from the Indian subcontinent (100), Middle East (nine), Far East (11), Africa (36) and Afro-Caribbean (four) in Cardiff, Wales published in 2002.^33^ Outcomes measured include maternal weight gain, vitamin D deficiency and baby weight.^24 33^ These interventions targeted the patient level of the framework with improved weight gain in mothers. In the 1981 study, daily weight gain (g/day) was higher in the interventions group (control group 46.4 (SD 29.5) vs intervention group 63.3 (SD 20.7), p<0.001) and in the 2002 study, reduced vitamin D deficiency was observed postdelivery (mean vitamin D level at booking vs postdelivery (5.79 (SD 0.91) vs 11.24 (SD 6.34))^24 33^ (online supplemental file 9). However, a lack of comparison with other ethnic groups may limit inference on the potential to reduce ethnic health inequalities.

### Interventions with the potential to reduce ethnic health inequalities

**Figure 3:**
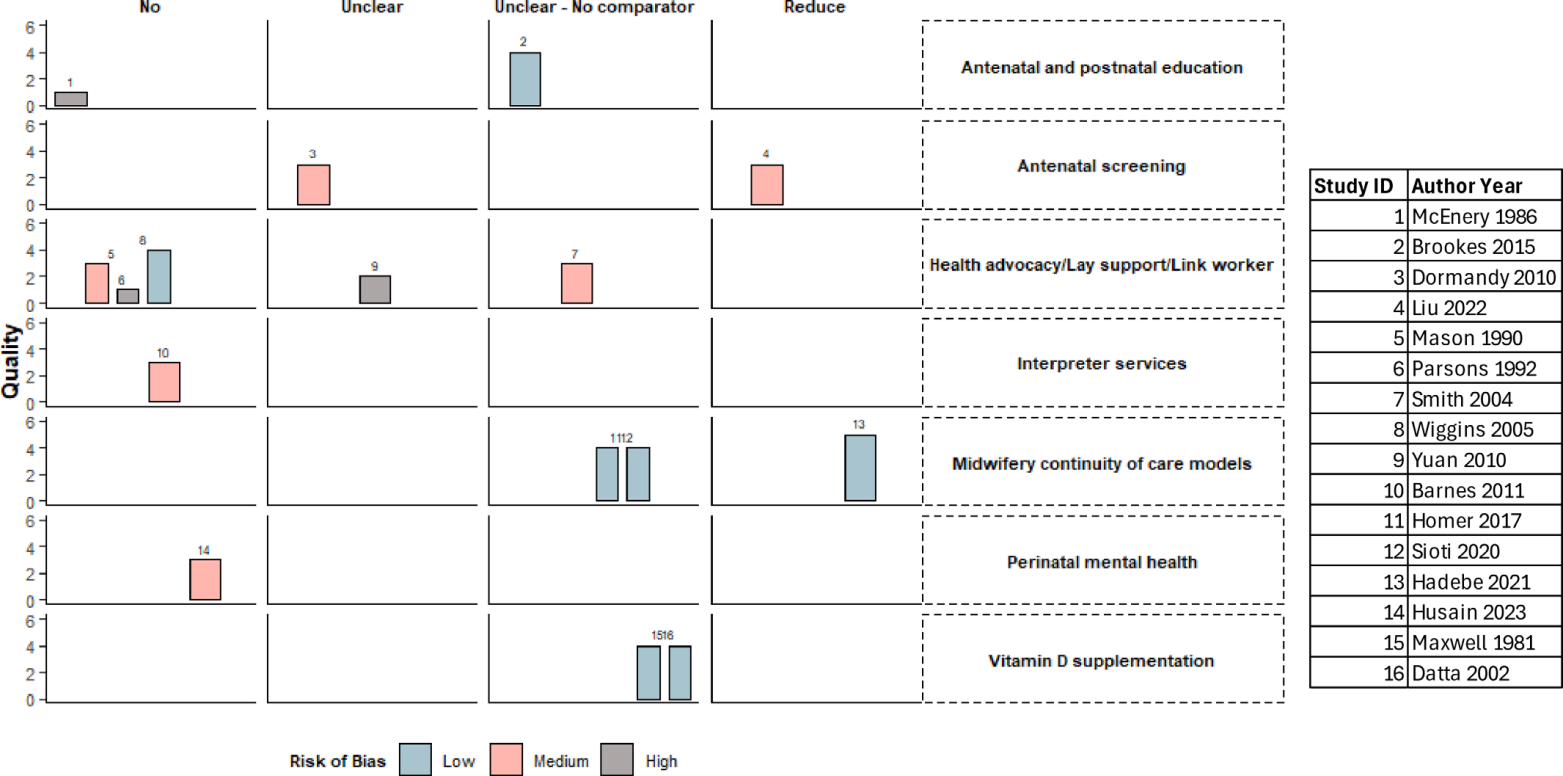
Harvest plot of identified interventions to reduce ethnic health inequalities in maternal, neonatal and child health. The figure shows the harvest plot of interventions with the potential to reduce ethnic health inequalities in maternal, neonatal and child health. The height of the bars represents the Mixed Methods Appraisal Tool quality criteria, and the study numbers are listed above the bars.

Based on the SWIM guidelines and vote counting of reported effect estimates for quantitative studies,^23^ two studies with MMAT quality rating of 60% and over demonstrated the potential to reduce ethnic health inequalities in maternal and infant health. They include: first-trimester screening for pre-eclampsia^31^ and MCoC—with the same team of midwives (figure 3 and online supplemental file 9). A further six studies with good outcomes but weak evidence for reducing ethnic health inequalities include MCoC—with the same midwife^29^ and for refugees and asylum seekers,^39^ Baby Steps parental education programme,^37^ link worker for weaning for British Pakistani women,^36^ social support for Chinese women^34^ and vitamin D supplementation.^33^

## Discussion

This systematic review identified 16 intervention studies that aimed to reduce ethnic health inequalities in maternal and neonatal health in over 40 years in the UK. Reported outcomes include uptake of early antenatal sickle cell screening, vitamin D deficiency, mode of delivery, place of birth, birth weight, stillbirth, preterm birth, infant bonding and weaning at 12 months. Mapping interventions onto a priori conceptual framework revealed a prevailing emphasis on patients in maternal health research, with only two interventions at the policy level. This promotes a ‘broken bodies’ discourse. This is due to an emphasis on individual risk factors with a lack of focus on targeted comprehensive policies in addressing wider social determinants of health shaping ethnic inequalities in maternal, neonatal and infant health.^40^

Following the SWIM guidelines in using vote count for direction of effect and assessing risk of bias due to anticipated heterogeneity in study designs, outcomes and population,^23^ first-trimester screening for pre-eclampsia^31^ and MCoC—with the same team of midwives^30^ may have the potential to reduce ethnic health inequalities in outcomes. However, inferences from studies evaluating these interventions are limited by the lack of reporting on reducing ethnic health inequalities,^29 30^ the fidelity of the interventions, study size,^24 33 36 37 39^ use of broad ethnic categories,^30^ baseline measurement of inequalities^37^ and geographical coverage.^30 37 39^ Notwithstanding, MCoC-related interventions that mapped across all levels of the framework remain the most promising.

### Comparisons with other studies

Limited evaluations exist for interventions addressing ethnic inequalities in the UK maternal system. In a Cochrane review, MCoC models were found to reduce preterm births and fetal loss for all women as findings were not stratified by ethnicity.^41^ A more comprehensive review of targeted health and social care interventions for women at risk of inequalities highlights the importance of multifaceted approaches, including community-centred and culturally competent care for vulnerable populations.^42^ In contrast, recent UK trial data suggest MCoC may be more effective for low-risk pregnancies, indicating the need for targeted implementation based on risk profiles and further research.^43^

### Strengths and limitations of this review

This systematic review covered the entire spectrum of antenatal and postnatal care to capture all available evidence on interventions at various points along the patient pathway in maternal and neonatal/infant health in the UK. This comprehensive review employed an exhaustive search strategy across five electronic databases, along with a meticulous examination of backward and forward citations in identified studies. The conceptual framework, originally developed from the systematic review by Clarke *et al*, which focused on interventions spanning three decades in the USA, was adapted for the UK healthcare system.^44^ The findings revealed a predominant focus on patient and provider levels, with a dearth of interventions at the microsystem, organisation and policy levels, and a notable lack of emphasis on neonatal outcomes. While the specified framework levels may have inherent limitations and potential for misclassification errors, adjustments were made to align with the UK healthcare system. Despite the geographical focus of the review by Clarke *et al* on the USA, the sources of ethnic inequalities in healthcare were deemed applicable to the UK setting, where structural, institutional and interpersonal racism play contributory roles.^45 46^ Recognising these limitations, the review highlights the importance of capturing all the available evidence on interventions targeting ethnic health inequalities in maternal and neonatal health while acknowledging potential reporting bias in favour of successful interventions.^15^

### Methodological limitations of included studies

The review period and the inclusion of different study designs meant that heterogeneity in the reporting of outcomes precluded a meta-analysis. Moreover, reporting of ethnicity in the included studies varied from large aggregations of White versus non-White to the five categories of White, Black, Asian, mixed and other. Some study authors were able to use the 16-level Office of National Statistics (ONS) ethnic reporting, while others used groupings to reflect migration patterns—such as African Indian or East Asian—and some used country of birth as the primary categorisation. For some of the studies, the data did not permit further disaggregation beyond White versus non-White to prevent deductive disclosure due to small sample sizes.

Finally, not all of the studies explicitly stated a reduction of ethnic inequalities in their objectives, provided baseline measures of inequalities, reported intervention fidelity and lacked a comparator. These elements are crucial for understanding how intervention planning and delivery have impacted the reduction of inequalities, and for identifying which group(s) the intervention is effective for.

### Implications for policy and practice

Despite the centrality of personalised care across all constituent countries of the UK,^47^,^50^ there is an urgent requirement for dedicated policies addressing ethnic health inequalities, particularly focusing on Black, Asian and minority ethnic women. Such policies are imperative to eliminate unjust practices within the UK’s maternal system. Currently, Better Births in England has a policy that 75% of Black, Asian and minority ethnic women within local maternity systems should have received midwifery-led continuity of care by 2024. However, the target was paused in September 2023 due to low staffing levels.^51^ While in Northern Ireland and Scotland, there is anticipation for a renewed policy following the expiration of the most recent policy in 2018^52 53^ and 2022, respectively.

This review identified a predominant focus on patient-level interventions, with only three of the continuity of care models addressing all framework levels and one potential to reduce ethnic health inequalities.^29 30 39^ Patient-focused approaches alone are insufficient for achieving equitable outcomes, as they fail to address structural healthcare barriers at policy and community levels. Given that health inequities extend beyond healthcare, an intersectional approach, examining the interplay of race, gender and socioeconomic factors, can inform integrated care systems’ development of place-based interventions that better respond to communities’ complex, overlapping needs.^54^

For meaningful intersectional analyses, granular ethnicity information is paramount. The review revealed significant variation in ethnicity reporting, from binary White/non-White classifications to detailed ethnic subgroups. This reflects the evolution from provider-assigned to self-identified ethnicity recording in healthcare settings.^55^ While small sample sizes often necessitated broader categorisations, upcoming initiatives like the NHS Digital Data Quality Maturity Index may enhance ethnicity data quality.^56^ Improved cross-sectoral data collection will enable more robust intersectional analyses of ethnic health inequalities’ multiple determinants.

Economic constraints have historically hindered consistent policy implementation, resulting in fragmented care practices despite existing national guidelines, as seen with vitamin D supplementation and interpretation services.^57^,^59^ While improved supplementation in some regions demonstrates potential benefits, progress is limited by the absence of a universal, equity-driven implementation framework.^58 60^ Many included studies were small-scale local evaluations with unclear wider implementation and variable reporting of intervention fidelity.^34 36^ This highlights the need for mandatory reporting of interventions targeting ethnic inequalities, facilitating effective dissemination and replication of successful initiatives across maternal and neonatal services through appropriate implementation models.^15^

### Implications for research

The review revealed critical evidence gaps, with only three policy-level interventions identified, highlighting limited influence on national regulations and resource allocation.^29 30 39^ Key challenges include evaluating simultaneous policy rollouts, insufficient focus on neonatal outcomes and absence of antiracism interventions addressing institutional and interpersonal racism. The lack of follow-up evaluations for small-scale initiatives suggests potential publication bias towards larger interventions.^15^

A disproportionate number of identified interventions specifically targeted South Asian pregnant women and mothers^24^,^2632 35 36^ with limited interventions targeting other minority ethnic groups, especially the Black African and Black Afro-Caribbean groups^28^ who have the highest maternal and perinatal morbidity and mortality. Effective interventions can be culturally adapted for women from underserved Black ethnic groups through co-production of interventions with service users who are most affected at the community level.

Geographical inequalities are apparent, with over a third of the included studies being heavily concentrated in London,^2425 27 29^,^31 61^ presenting challenges in extrapolating findings to other parts of the UK. This concentration raises concerns about the broader applicability of results to areas outside London, considering potential differences in migration patterns, local policies, community-based support and the distinct role of structural racism in regions with lower ethnic diversity.^62^

Despite the WHO 2003 recommendation for culturally appropriate interventions, only one study evaluated culturally adapted CBT for South Asian women. While this intervention showed no significant improvement in postnatal depression, earlier pilot studies demonstrated participant acceptance and mental health benefits. A larger multisite trial is ongoing and due to publish findings.^63^

Despite long-standing evidence of poorer experiences among ethnic minority patients in obstetric services,^57 64^ no included studies explicitly addressed racism. Implementing antiracism interventions remains challenging due to their complex, multicomponent nature. Evidence suggests Black, Asian and minority ethnic women may internalise experiences of racism, with cultural and religious factors potentially inhibiting reporting and redress.^15^

## Conclusion

Evidence for interventions addressing ethnic health inequalities in UK maternal and neonatal health remains limited, with most targeting patient-level rather than structural policy changes. While some interventions show promise, future studies should incorporate conceptual frameworks with clear levers of change as adopted in this study, clear ethnicity classification, baseline inequality measurements and explicit objectives targeting ethnic health inequalities. These findings can guide policymakers and commissioners in developing co-designed interventions that ensure proportionate universalism while addressing current research gaps.

## Supplementary material

10.1136/bmjph-2024-001476 online supplemental file 1

10.1136/bmjph-2024-001476 online supplemental file 2

10.1136/bmjph-2024-001476 online supplemental file 3

10.1136/bmjph-2024-001476 online supplemental file 4

10.1136/bmjph-2024-001476 online supplemental file 5

10.1136/bmjph-2024-001476 online supplemental file 6

10.1136/bmjph-2024-001476 online supplemental file 7

10.1136/bmjph-2024-001476 online supplemental file 8

10.1136/bmjph-2024-001476 online supplemental file 9

## Data Availability

All data relevant to the study are included in the article or uploaded as supplementary information.
