# Supplementary material for: Systematic review of interventions to reduce ethnic health inequalities in maternal and perinatal health in the UK
Source: BMJ Public Health. 2025 Jul 15;3(2):e001476. doi: 10.1136/bmjph-2024-001476 (PMC12273135; doi:10.1136/bmjph-2024-001476)
Supplement: online supplemental file 2 [file bmjph-3-2-s002.docx]

**Supplementary File 2. Inclusion and exclusion criteria for studies**

| **Criteria** | **Inclusion** | **Exclusion** |
| --- | --- | --- |
| **Date** | Published since database inception up to 11^th^ August 2023. | None |
| **Setting** | Any healthcare setting in the UK. | 1. Study conducted outside of the UK, or it is a multi-country study excluding data from the UK. 2. A non-healthcare setting . |
| **Population** | 1.Outcome must be specific in at least one of the following groups: pregnant women, post-partum women, babies (of any gestational age), infants up to the age of .  2. Ethnicity can be a place of birth, nationality, and migration status and can be self-reported or based on reporting by health care records. This may be aggregated or disaggregated.  3. At least one ethnic minority group specified. | 1. No ethnic minorities specified or no information on ethnicities involved in the study.   2. Study outcomes not specific to pregnant women, post-partum women, babies (of any gestational age) or infants up to the age of 1. |
| **Intervention** | 1.An evaluation or assessment of an intervention or healthcare access   2. Intervention may include a trial, programme, implementation, service or specific policy.  3.Intervention must be specific to improve outcomes in maternal and/or neonatal health such as health promotion or identifying unmet needs, therefore reducing maternal and/or neonatal health inequalities. | 1. Intervention was not specific to improve outcomes in maternal and/or neonatal health such as health promotion or identifying unmet needs, therefore reducing maternal and/or neonatal health inequalities.   2. Experiences of services for ethnic minority groups or access to services without intervention or policy.   3. Observational studies on health outcomes such as mortality and morbidity without an intervention. |
| **Comparator** | Studies may include a comparison of outcomes between ethnic groups, but this is not compulsory. |  |
| **Outcomes** | 1.The main outcomes of interest are the impact of the intervention on maternal newborn and/or infant health inequalities amongst ethnic minority groups in relation to the National Maternity and Perinatal Audit outcomes (e.g. antenatal care coverage including genetic screening, stillbirths, gestational age, birthweight, small for gestational age at birth, maternal and infant mortality, immunization, birth attendance, breastfeeding).  2. Outcome measures include a report on ethnic inequality in outcomes (e.g. differences in outcomes for women and or infants by ethnic group) or any other measures used by authors for measuring a change in inequalities after interventions (e.g. access to care, quality of care, engagement with services, experiences and health outcomes).  3. Studies will be included irrespective of whether the intervention has been identified as a success, or not, to help meet the objectives of this review.  4.Studies that focus on issues commonly associated with minority ethnic groups (such as sickle cell diseases, thalassaemia, hepatitis B, and tuberculosis). | 1. No ethnicity, place of birth, nationality, or migration status was reported.    2. Outcomes not specified by ethnicity either aggregated or disaggregated.  3. Studies on general maternal or perinatal interventions if they do not report outcomes by ethnicity. |
| **Outcomes** | 1. An evaluation or assessment of an intervention or healthcare access   2. Intervention may include a trial, programme, implementation, service or specific policy.   3. Healthcare access may include quality of care, healthcare disparity and socioeconomic inequalities.   4. Intervention must be specific to improve outcomes in maternal and/or neonatal health such as health promotion or identifying unmet needs, therefore reducing maternal and/or neonatal health inequalities. | 1. Did not include an evaluation or assessment of an intervention or health care access.    2. Intervention was not specific to improve outcomes in maternal and/or neonatal health such as health promotion or identifying unmet needs, therefore reducing maternal and/or neonatal health inequalities.   3. Experiences of services for ethnic minority groups or access to services without intervention or policy.   4. Observational studies on health outcomes such as mortality and morbidity without an intervention. |
| **Study Design** | Systematic reviews, randomised controlled trials (RCTs), observational studies (cohort, cross-sectional, audit), qualitative studies (interviews, focus groups), and mixed methods studies. | Non-original studies, such as opinion papers, and commentary. |
| **Publication Type** | Academic journal articles, and reports from organisations. | Books or book chapters, blogs, any publication other than study reports in a peer-reviewed journal or from an organisation |
| **Language** | English Language | Papers not published in the English Language |
