# Supplementary material for: Systematic review of interventions to reduce ethnic health inequalities in maternal and perinatal health in the UK
Source: BMJ Public Health. 2025 Jul 15;3(2):e001476. doi: 10.1136/bmjph-2024-001476 (PMC12273135; doi:10.1136/bmjph-2024-001476)
Supplement: online supplemental file 4 [file bmjph-3-2-s004.docx]

**Supplementary File 4. Conceptual framework definitions**

| **Conceptual framework themes** | **Definitions** |
| --- | --- |
| Patient | Change knowledge and/or behaviours  of patients and/or their family members or partners (e.g. self- management) |
| Provider | Change the knowledge and/or behaviour of providers (e.g. identifying language barriers) |
| Microsystem (Immediate care team) | Add new members to, or shift  care team, such as the primary care provider, nurse, and staff (e.g. integrate peer educators into the care team |
| Organisation | Change organization operations involving, but not limited to, clinic flow, information technology, and/or human resources (e.g. introducing Electronic Medical Records |
| Community | Work with community organizations  and/or community members who are not established patients at the intervening organization (e.g. health education for specific ethnic groups or religious group) |
| Policy | Influence laws, regulations, and/or  resource allocation on a regional or national basis (e.g., national targets) |
