# Supplementary material for: Systematic review of interventions to reduce ethnic health inequalities in maternal and perinatal health in the UK
Source: BMJ Public Health. 2025 Jul 15;3(2):e001476. doi: 10.1136/bmjph-2024-001476 (PMC12273135; doi:10.1136/bmjph-2024-001476)
Supplement: online supplemental file 6 [file bmjph-3-2-s006.docx]

**Table 1. Characteristics of included studies with information on the intervention for each study**

| **Author Year** | **UK/NHS England Region** | **Study Design;Data Source** | **Sample size (age in years);[dates data collection]** | **Ethnic minority group;(% of total sample)** | **Population** | **Intervention** | **MMAT score** |
| --- | --- | --- | --- | --- | --- | --- | --- |
| **Antenatal and Postnatal Education** | | | | | | | |
| McEnery 1986 ^26^ | London, England | Cohort | 69 (18-40); [1980-1982] | Pakistani, East African Asians | Mothers and infants | A course of 12 weekly lectures, each lasting 11/2 hours, covering fertility, pregnancy, childbirth and childrearing by a health visitor, midwife or nutritionist to experimental group | * |
| Brookes 2015 ^38^ | West Midlands, England | Qualitative; Primary data collection | 14 parents(3 fathers, 11 mothers), | Pakistani, Indian, Chinese, Oromo(Cultural group in Ethiopia), Somali, Bangladeshi, Caribbean(St Vincent) | Parents | Perinatal education programme, Interpreter services | **** |
| **Antenatal Screening** | | | | | | | |
| Dormandy 2010 ^29^ | London, England | RCT; Other | 1454(>18);[2005-2006] | North European, South or Southeast Asian, African/Caribbean, South European, Other and Mixed; (60.3%) | Pregnant women and Fathers | Universal antenatal sickle cell and thalassaemia (SCT) screening in primary care when pregnancy is first confirmed. | *** |
| Liu 2022 ^32^ | London, England | Cohort; Hospital records | 20651(NICE: 32.6 (29.2-35.9); FMF 32.8(29.4-35.9));[2016-2020] | White, Black, Asian and Mixed/Other; (34.4%) | Pregnant women | First-trimester screening algorithm for placental dysfunction | *** |
| **Health Advocacy/Lay Support/Link worker** | | | | | | | |
| Mason 1990 ^36^ | East Midlands, England | Case-control; Primary data collection | 485(NR);[1985-1986] | Asian women; (100%) | Pregnant women, Mothers and Babies | Asian Mother and Baby Campaign - Link workers | **** |
| Parsons 1992 ^27^ | London, England | Unknown; Hospital records | 1000(NR);[1986] | Born in Asia or Turkey; (100%) | Pregnant women | Multiethnic Women's Health Project- Health advocacy | * |
| Smith 2004 ^37^ | East of England, England | Qualitative; Other | 30(3 months);[2000] | British Pakistani; (100%) | Babies | Weaning intervention delivered via link workers | ***** |
| Wiggins 2005 ^28^ | London, England | RCT; Primary data collection | 731 (NR);[1999] | Women living in deprived areas Camden and Islington; (42.5%) | Mothers | Postnatal support | **** |
| Yuan 2010 ^35^ | Belfast, Northern Ireland | Quasi-experimental | 32 (NR); (NR) | Chinese (100%) | Mothers | Social support for bonding through oral health education | *** |
| **Interpreter Services** | | | | | | | |
| Barnes 2011 ^39^ | England | Mixed methods | 1304(<16 -24);[2007-2009] | White, Black, Asian , Mixed or Other (18.9%) | Mothers, Nurses and Interpreters | Interpreters to deliver Family-Nurse Partnership | ** |
| **Midwifery continuity of care (MCoC)** | | | | | | | |
| Homer 2017 ^30^ | London, England | Cohort; Primary data collection | 2568(14-50);[1997-2009] | Black African, Black Caribbean, Black British, Asian, Mixed, Other and Unknown (63.1%) | Mothers and Babies | Albany Midwifery practice model (Antenatal and Postnatal visits and support including home visits) | **** |
| Sioti 2020 ^40^ | Yorkshire & The Humber | Mixed method evaluation | 21(18-34); [Feb 2018-Feb 2019] | Democratic Republic of Congo, Eritrea, India, Iran, Kurdistan, Kuwait, Nigeria, Pakistan, Saudi Arabi, Sri Lanka, Sudan, Syria, Yemen. | Mothers and infants | ORAMMA - Model of care for migrant, asylum-seeking and refugee women (healthcare, social care support and peer support) | **** |
| Hadebe 2021 ^31^ | London, England | Cohort;Hospital records | LEAP Area Caseload: 230 pregnancies, LEAP traditional care: 293, NON LEAP All care : 8,430 pregnancies (NR);[2018 - 2020] | White, Black, Asian, Mixed, Other;(LEAP - 47.8%, LEAP traditional - 42.8%, NON LEAP All care - 37.8%) | Mothers and Babies | Targeted caseload midwifery | ***** |
| **Perinatal mental health intervention** | | | | | | | |
| Husain 2023 ^33^ | North West | RCT; Primary data collection | 83 (>16); [ ] | Indian, Pakistani, Bangladeshi; (100%) | Mothers | Culturally-adapted cognitive behavioural therapy based intervention (Positive Health Programme) for maternal postnatal depression. | *** |
| **Vitamin D** | | | | | | | |
| Maxwell 1981 ^25^ | London | RCT; Primary data collection | 126(NR);[NR] | Asian; (100%) | Pregnant women | Supplementary vitamin D administered in the last trimester of pregnancy to Asian women. | **** |
| Datta 2002 ^34^ | Wales | RCT; Primary data collection | 160 (NR); [1995-1996] | 100 from Indian subcontinent, 4 were Afro-Caribbean, 9 from Middle East, 11 from Far East, 36 from Africa | Pregnant women | Biochemical screening of women from ethnic minorities in early pregnancy and subsequent supplementation and rechecking status | **** |

MMAT – Mixed Methods Appraisal Tool. Star rating is used to prevent from a focus on a single score ^22^.
