# Supplementary material for: Systematic review of interventions to reduce ethnic health inequalities in maternal and perinatal health in the UK
Source: BMJ Public Health. 2025 Jul 15;3(2):e001476. doi: 10.1136/bmjph-2024-001476 (PMC12273135; doi:10.1136/bmjph-2024-001476)
Supplement: online supplemental file 9 [file bmjph-3-2-s009.docx]

**Supplementary File 9. Characteristics of interventions, outcomes and potential to reduce ethnic inequalities.**

| **Author Year** | **Ethnic minority group** | **Intervention** | **Comparator** | **Adjustment** | **Outcome** | **Reduced ethnic inequalities** |
| --- | --- | --- | --- | --- | --- | --- |
| **Antenatal and Postnatal Education** | | | | | | |
| McEnery 1986^26^ | Pakistani, East African Asians | Antenatal education | Normal ranges for laboratory tests; data on Caucasian children | No | Breastfeeding, APGAR score, birthweight, infant health | Unclear |
| Brookes 2015^38^ | Pakistani, Indian, Chinese, Oromo(Cultural group in Ethiopia), Somali, Bangladeshi, Caribbean(St Vincent) | Perinatal education programme | None | N/A | Based on the theory of change model for the Baby Steps programme the key outcomes include:   - Improved parent–infant relationship - Improved confidence and self-esteem - Improved couple relationship - Improved social support - Reduced symptoms of anxiety and depression - Improved knowledge about pregnancy, birth and child development   The main goals:   - Parents are better able to provide appropriate, consistent and sensitive care for their babies - Better birth outcomes   **Reported outcomes:**  Parents reported positive outcomes, including increased knowledge about pregnancy and parenting, improvements in relationships with partners and babies, and increased confidence. Baby Steps was seen as a particularly important source of information and support for parents who were not integrated into the local community, particularly refugees and asylum seekers. The study demonstrated that perinatal education may have the capacity to change attitudes and behaviour towards gender roles and abusive practices such as female genital mutilation. Factors that engaged minority ethnic parents in the Baby Steps programme included: the use of interpreters, cultural competence among practitioners, and practitioners working flexibly by offering additional support and making themselves available to liaise with other agencies. | Potential to reduce – limited by lack of a comparator |
| **Antenatal Screening** | | | | | | |
| Dormandy 2010^29^ | North European, South or Southeast | Early screening for sickle cell in primary care with parallel father testing (test offered to mother and father | Standard care | No | The estimated adjusted difference between the midwife care and GP parallel testing arms was 16.5% (95% confidence interval 7.1% to 25.8%; P=0.002) and between the midwife care and GP sequential testing arms was 27.8% (14.8% to 40.7%; P<0.001) | Unclear |
| Liu 2022^32^ | Cypriot, Indian, Pakistani and Italian | Fetal Medicine Foundation (FMF) first trimester screening programme for placental dysfunction | Standard care | No | Perinatal Deaths. White (Nice screened vs. FMF screened, odds ratio 0.969 (0.493-1.908)), Non-white ethnicities (Nice screened vs. FMF screened, odds ratio 0.403 (0.206-0.789)) | Potential to reduce – broad ethnic categories and no adjustment for confounders |
| **Health Advocacy/Lay support/Link worker** | | | | | | |
| Mason 1990^36^ | Asian | Asian Mother and baby campaign | a) community and hospital link workers; b) community link worker only; c) hospital link worker only; d) did not see a link worker (controls). | English proficiency | Birthweight data not shown. | No |
| Parsons 1992^27^ | North European, South or Southeast, Asian, African/Caribbean, South European, Other and Mixed | Multiethnic Women's Health Project- Health advocacy | Women who delivered at Mothers' Hospital before (1979) and after the intervention (1986), Women at Whipps Cross Hospital in 1986 and 1979 | No | Antenatal length of stay remained the same between the two time periods (5.9 vs. 5.8) in the reference hospital and decreased from 8.6 days to 5.7, P<0.001; CS increased from 11% to 1 7% per cent at the reference hospital, whereas they fell from 10.8% to 8.5% percent at the Intervention hospital, P<0.001. | No – high risk of bias |
| Smith 2004^37^ | British Pakistani | weaning intervention delivered via the role of the link worker | None | N/A | By age one, intervention objectives met: children should have a varied diet, family foods (92%); drinking juice/water from cup (100%), finger feeding (100%). | Potential to reduce – no comparator with other ethnic groups |
| Wiggins 2005^28^ | White, Black, Asian and Mixed/Other | Postnatal support | Standard services | Type of delivery, baby’s gender, ethnicity, mother’s education, existing support | At 12 and 18 months maternal smoking (SHV: 0.86; 0.62 to 1.19, CGS: 0.97; 0.72 to 1.33) or maternal depression (SHV:0.86; 0.62 to1.19, CGS: 0.93; 0.69 to 1.27. | No |
| Yuan 2010^35^ | Chinese | Chinese Immigrant Mothers oral health Education (ChIME) programme | Control group | No | At 12-month follow-up the intervention group compared with control group mothers had statistically significantly greater decreases in mean scores for the scales bonding disorder (-7.18, 95% CI -10.07,-4.18 vs. -2.41, 95% CI -4.48, -0.35), P=0.009; rejection and pathological anger (-4.82, 95%CI -7.32, -2.32 vs. -1.00, 95% CI -2.19, 0.19), P=0.006, and infant-centred anxiety (-3.00, 95%CI -4.57, -1.42 vs. -1.06, 95% CI -2.08, -0.04), P=0.04 | Unclear as oral health improvement was the aim |
| **Midwifery Continuity of Care (MCoC)** | | | | | | |
| Homer 2017^30^ * | Black African, Black Caribbean, Black British, Asian, Mixed, Other, Unknown | Albany Midwifery practice model (Antenatal and Postnatal visits and support, including home visits) | None | Age, parity, council housing | Outcomes for Black, Asian and Minority ethnic women vs. White women: Preterm birth ( < 37 weeks) 100 (6.2%) vs. 30 (3.2%) p= < 0.001; Low birth weight ( < 2500 g) 90 (6.1%) 28 (3.2%) 0.002 | Unclear – Study shows Black, Asian and Minority Ethnic women were more likely to have preterm or low birth weight babies than White women but insists the preterm and low birth weight were lower than the general population due to a lack of comparator and insists the practice model was effective for Black, Asian and Minority ethnic women |
| Sioti 2020^40^ | Democratic Republic of Congo, Eritrea, India, Iran, Kurdistan, Kuwait, Nigeria, Pakistan, Saudi Arabi, Sri Lanka, Sudan, Syria, Yemen. | Healthcare, Social care, Peer support. | None – Conducted across three European countries (UK, Greece and Netherlands) | No | UK results - APGAR scores above 7 in the 1st minute (16, 80%) and 5th minute (18, 90%); the average neonatal birth weight was 3400g, range 2260-4710, with two premature deliveries. At discharge from the hospital, 19 (95%) of the neonates were breastfeeding, of which 12 (60%) were breastfeeding exclusively.  The programme was evaluated as highly acceptable and satisfactory by the participants, the MAR  mothers, the MPSs and the HCPs. The cultural appropriate and individualized care provided by the  60 members of the ORAMMA multidisciplinary team, as well as the support provided by MPSs, was identified as a success factor for the in improving engagement as almost all women (95.2%, n=20) did not have previous contact with a health care professional during the current pregnancy until contact with the ORAMMA team. | Potential to reduce – limited by lack of a comparator |
| Hadebe 2021^31^ | White and Bangladeshi | Targeted caseload midwifery | Standard care | Inverse probability weighting on Interpreter needed, unknown ethnicity, respiratory comorbidity, previous instrumental birth | **Greater reduction in caesarean rates in** **Black, Asian and Minority ethnic women** (43.1% -27.8%); Risk Ratio – 0.68 (0.47 to 0.99) P-value 0.04 vs. white women (39.8%-24.7%); Risk Ratio – 0.63 (0.40 to 0.99) P-value 0.0.04 compared with standard care.  **Preterm birth rate reduced by ~half in Black, Asian and Minority ethnic women** (14.4% -7.3%); Risk Ratio – 0.49 (0.21 to 1.09) P-value 0.08 vs. white women (5.1%-2.5%); Risk Ratio – 0.45 (0.08 to 2.31) P-value 0.23 compared with standard care.  **Marked reduction in births under 34 weeks in Black, Asian and Minority ethnic** women (7.2% -1.8%); Risk Ratio – 0.66 (0.07 to 7.2), P-Value 0.7 vs. white women (2.0%-1.2%); Risk Ratio – 0.24 (0.05 to 1.12) P-Value 0.07.  **Women who needed an interpreter**  There was a statistically significant reduction in preterm birth rate (before 37 weeks) in those allocated to caseload midwifery compared with traditional care, with a greater impact in those who needed an interpreter (5.3% vs 44.4%; risk ratio: 0.11; p=0.03; 95% CI 0.01 to 0.83). compared with those who did not need an interpreter (5.6% vs 9.2%). | Potential to reduce |
| **Interpreter Services** | | | | | | |
| Barnes 2011^39^ | British Pakistani | Interpreters to deliver Family-Nurse Partnership | No interpreter | No | Quantitative indicators: The percentage of planned content covered in visits was lower with interpreters (pregnancy 90% vs. 94%; infancy 88% vs. 93%). Qualitative: Some nurses and clients reported that they would rather manage without an interpreter. | No |
| **Perinatal mental health** | | | | | | |
| Husain 2023^33^ | Indian, Pakistani, Bangladeshi | Culturally-adapted cognitive behavioural therapy based intervention (Positive Health Programme) for maternal postnatal depression. | Treatment as usual (TAU) | English proficiency | Severity of depression, satisfaction with treatment, measure of relationship adjustment, perceived social support, sources of stress in family and assessment of symptoms of depression through clinical interviews. | Unclear |
| **Vitamin D Supplementation** | | | | | | |
| Maxwell 1981^25^ | Asian women | Vitamin D intervention | Asian women without Vitamin D intervention | No | Maternal data: average daily weight gain in last trimester (g/day)- Control 46.4 (SD 29. 5) vs. Intervention arm 63.3 (SD 20.7), P<0.001. Infant data: Mean birth weight (g) - Control 3034 (SD 524), Intervention arm 3157 (SD 469), P>0.05;No. with birth weight under 2500 g; Control 15, Intervention arm 7, P >0.05 | Unclear – no comparator with other ethnic groups |
| Datta 2002^34^ | 100 from Indian subcontinent, 4 were Afro-Caribbean, 9 from Middle East, 11 from Far East, 36 from Africa | Vitamin D supplements | At booking vs post-delivery | N/A | Mean vitamin D level at booking - 5.79 (SD 0.91). Mean vitamin D level post-delivery - 11.24 (SD 6.34) | Unclear - no comparator with other ethnic groups |

N/A – Not applicable; SD – Standard Deviation
